# A Novel Approach Endowing Nanocalcium Carbonate with Amphiphobic Properties Through Perfluoropolyether Carboxylic Acid Surface Modification: Elucidating the Surface Modification Mechanism

**DOI:** 10.3390/molecules31183320

**Published:** 2026-09-19

**Authors:** Jincai Zhang, Wanru Han, Ding Meng, Vishnu Vijay Kumar

**Affiliations:** 1 Institute of Resources and Environmental Engineering, Shanxi University, Taiyuan 030006, China; h13571176237@163.com (W.H.); chanerme@126.com (D.M.); 2 State Environmental Protection Key Laboratory of Efficient Utilization of Waste, Taiyuan 030031, China; 3 National Engineering Research Center of Solid Waste Resource Recovery, Taiyuan 030031, China; 4 Faculty of Engineering, Higher Colleges of Technology, Abu Dhabi 25035, United Arab Emirates; vishnu.vijay@u.nus.edu

**Keywords:** nanocalcium carbonate, surface modification, perfluoropolyether carboxylic acid, reaction mechanism

## Abstract

Acquiring an amphiphobic nanocalcium carbonate (NCC) is a perplexing problem in the long term because of its hydrophility. This restricts its application in self-cleaning coatings and other super-low surface energy engineering fields. This study offers a novel and simple approach that endows NCC with an amphiphobic property through surface modification using perfluoropolyether carboxylic acid in the presence of sodium hydroxide. The modification process was optimized at a 5 wt% modifier concentration, 50 °C, and a duration of 40 min. Comprehensive characterization was performed using FT-IR, TG-DTA, SEM, HR-TEM, NMR and XPS techniques, in contrast with the previous literature describing hydroxyl groups. Notably, FT-IR analysis indicates that no hydroxyl groups were detected on the NCC surface. The modification mechanism involves COO^−^ groups from the modifier forming Ca-O ionic bonds with calcium sites on the NCC surface. HR-TEM analysis revealed an exceptionally thin modifier film (2–5 nm) on the NCC surface. Despite this minimal thickness, it endows NCC with remarkable amphiphobic properties, with water and oil contact angles up to 120.4° and 139.9°, respectively. These findings expand insights into the surface modification mechanism of NCC and its potential application fields.

## 1. Introduction

Nanocalcium carbonate (NCC) exhibits superior physicochemical properties compared with those of conventional calcium carbonate, particularly for electrical, optical, and mechanical applications [1]. Its widespread availability, cost-effectiveness, excellent whiteness, chemical inertness, nonflammability, and low oil and water absorption characteristics make it a versatile filler in various industries, including plastics, rubber, paper, paint, and ink [2]. NCC typically occurs in three crystalline polymorphs: calcite, vaterite, and aragonite [3,4,5]. These polymorphs exhibit diverse morphologies, such as cubic, spherical, columnar, and other distinctive shapes [6,7,8], further expanding their potential uses. The inherent hydrophilicity of NCC facilitates its integration into water-based systems. Therefore, it is commonly used as a filler in aqueous coatings, inks, and other water-based products. However, when it is used in an organic matrix such as plastics or resin composites, the necessary step to take in advance is surface modification; this ensures good interfacial compatibility between the filler and matrix and further improves the overall composite performance. This modification process alters the surface properties of NCC, enabling its effective incorporation into a broader range of materials and expanding its functional capabilities.

For application in an organic matrix, NCC requires a lipophilic surface that can form a strong bond with the organic matrix, thereby yielding high-performance composite materials. A wide range of modifiers have been employed for this purpose, including sodium stearate [9], sodium oleate [10], stearic acid [11], various fatty acids [12,13], hydroxylated polyvinyl chloride [14], lauric acid [15], and poly(methyl methacrylate) [16]. In addition to their traditional role as composite fillers, these modified NCCs (MNCCs) have found novel applications in diverse fields. Recent research has explored their potential for improving suspension stability for biological applications [17], enhancing Cu^2+^ removal in environmental remediation [18], and optimizing lubricating oil performance [19]. The past two decades have witnessed significant research interest and rapid advancements in this area. Scientists and industry professionals have recognized the versatility and potential of MNCCs, leading to extensive investigations and developments across multiple sectors. This growing body of research continues to expand our understanding of NCC’s capabilities and opens new avenues for its application in both established and emerging fields.

Current surface modification methods for NCC typically impart either hydrophobic [20] or oleophobic properties, but rarely both. As a result, the resulting surfaces are susceptible to fouling by either oils or aqueous liquids. There is a notable scarcity of modifiers capable of imparting amphiphobic characteristics to NCC, i.e., characteristics that make the surface repels both water and oil. These amphiphobic inorganic powder surfaces could have significant applications in antifogging films, self-cleaning coatings, and antibioadhesion coatings [21]. This gap in NCC modification technology presents an opportunity for innovative research to develop truly amphiphobic surfaces, potentially expanding its application in the aforementioned fields.

Achieving amphiphobic characteristics in NCC requires coating or grafting its surface with a material of extremely low surface energy. Our previous research demonstrated that pitch fluorides exhibit exceptionally low surface energy (2.0 mJ/m^2^), which is lower than those of other commonly recognized low surface energy materials such as graphite fluoride (7.0 mJ/m^2^) and polytetrafluoroethylene (15.88 mJ/m^2^) [22,23]. This property is primarily attributed to the high concentration of CF_3_ and CF_2_ groups in these molecules. However, pitch fluorides lack the functional groups necessary to react directly with NCC, presenting a challenge for effective surface modification. This limitation necessitates the exploration of alternative approaches for coating or grafting these low-surface-energy materials onto NCC surfaces. Sawada et al. [24] reported the fabrication of perfluoropolythiophene/dicarboxylic acid/silica nanocomposites exhibiting superoleophobic and superhydrophilic properties. Their approach utilized perfluoropolycyclic dicarboxylic acid, which features a CF_2_ and CF_3_ main chain with both terminal -COOH groups. One -COOH group reacted with surface -OH groups on silica, while the other remained exposed, contributing to the superhydrophilic nature of the material. However, this study did not conclusively demonstrate amphiphobicity. This research highlights the complexity of achieving true amphiphobicity in inorganic powder materials and indicates that amphiphobicity modification remains a challenge.

This study investigates the use of perfluoropolyether carboxylic acid (PFPECA) to modify NCC, aiming to create an amphiphobic surface. PFPECA, with its single terminal -COOH group, is structurally similar to perfluoropolycyclic dicarboxylic acid. We explore the modification mechanism and process in detail, focusing on achieving both hydrophobic and oleophobic properties. To our knowledge, this is the first report on the use of PFPECA to impart amphiphobic characteristics to NCC, potentially opening new avenues for advanced material applications.

## 2. Results and Discussion

### 2.1. Optimization of Modification Conditions

**Figure 1:**
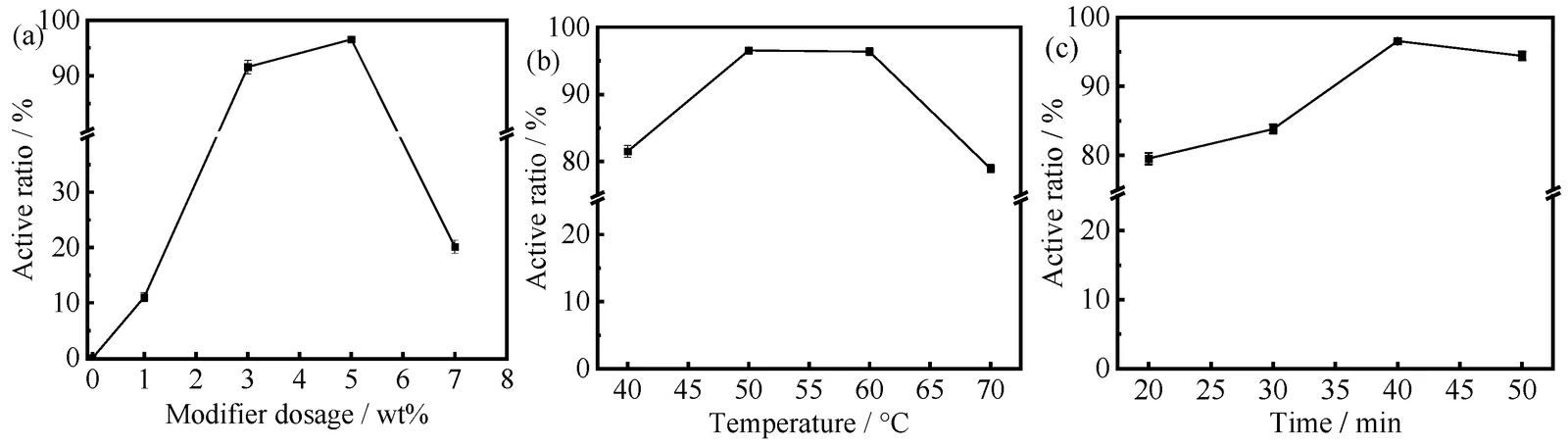
Effect of modification conditions on the active ratio. (**a**) PFPECA dosage (**b**) Temperature (**c**) Time.

The changes in the surface zeta potential and active ratio of inorganic powders are two important indicators of the effect of surface modification. First, to investigate the effect of modifier dosage on the active ratio of MNCC, modification reaction was conducted under the following conditions: 50 °C, 40 min and varying modifier dosages. The results indicate that as the the modifier dosage increases, the active ratio first increases, reaching a maximum of 96.55% at a PFPECA content of 5 wt%, and then decreases, as shown in Figure 1a. Second, with the modifier dosage fixed at 5 wt% and the modification time fixed at 40 min, the effect of modification temperature on active ratio was investigated. Figure 1b shows that the active ratio exhibits a three-stage changes: the active ratio increase as temperature increases in the range of 40 to 50 °C; then remains a nearly constant value in the temperature range of 50 to 60 °C, finally it drops in the range of 60 to 70 °C. Third, with the PFPECA dosage fixed at 5 wt% and the modification temperature fixed at 50 °C, the effect of reaction time on active ratio was investigated. The results indicate that as time gose on, the active ratio first gradually increases from 20 to 40 min, and then slowly decreases from 40 to 50 min. At 40 min, it also reaches the maximum value of 96.55% (Figure 1c). The investigation of the active ratio demonstrates that the suitable modification conditions are as follows: 5 wt% PFPECA, 50 °C and 40 min. An active ratio of 96.55% indicates that the NCC surface was almost completely converted from hydrophilic to hydrophobic.

**Figure 2:**
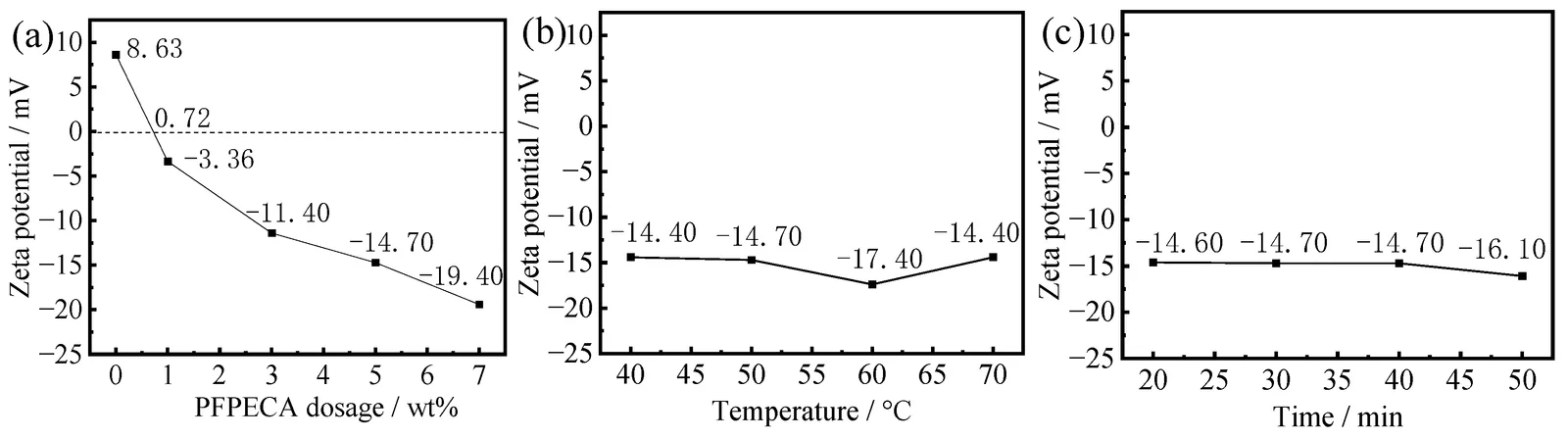
Effect of modification conditions on the zeta potential of sample surfaces. (**a**) modifier dose, (**b**) temperature, and (**c**) time. (0.72 wt% is the charge transition point in (**a**)).

The zeta potential represents the charge carried in the sliding shear layer at the particle surface. The polarity of the charge is usually the same as that of the particle surface charge and opposite to that of the charge carried by the middle Stern layer. Whether the charge is positive or negative, the greater charge number, the higher the zeta potential of particle, and the more easily the particles are stably dispersed in aquesous solution. In other words, a high absolute value of the zeta potential can keep the particles uniformly and stably dispersed in solution when they are applied in aqueous coatings, thus achieving optimal coating performance [25,26]. In this study, following the same method as described above, the effect of PFPECA dosage, temperature and time on the zeta potential were investigated. The results indicate that the modification reaction dramatically converted the zeta potential from positive (8.63 mV for NCC) to negative (−19.4 mV for MNCC) as the modifier dosage increased from 0 wt% to 7 wt% (Figure 2a). Reaction temperature and time had almost no effect on zeta potential (Figure 2b,c). The above results demonstrate that the key factor determing the zeta potential change is the modifier and its dosage. The zeta potential of unmodified NCC is positive, originating from the Ca^2+^ ions on its surface. After modification, the zeta potential of MNCC is negative, possibly resulting from the -COO^−^ groups at the terminals of PFPECA molecules after dehydrogenation. Combined with the influence results of the above active ratio, the optimal modification conditions remain the same as above.

### 2.2. Analysis of NCC and MNCCs

The above results indicate that the modification reaction has converted the surface properties of NCC. To investigate the reason for this, FT-IR, SEM and HR-TEM were used to characterize the NCC and MNCCs containing varying modifier dosages.

**Figure 3:**
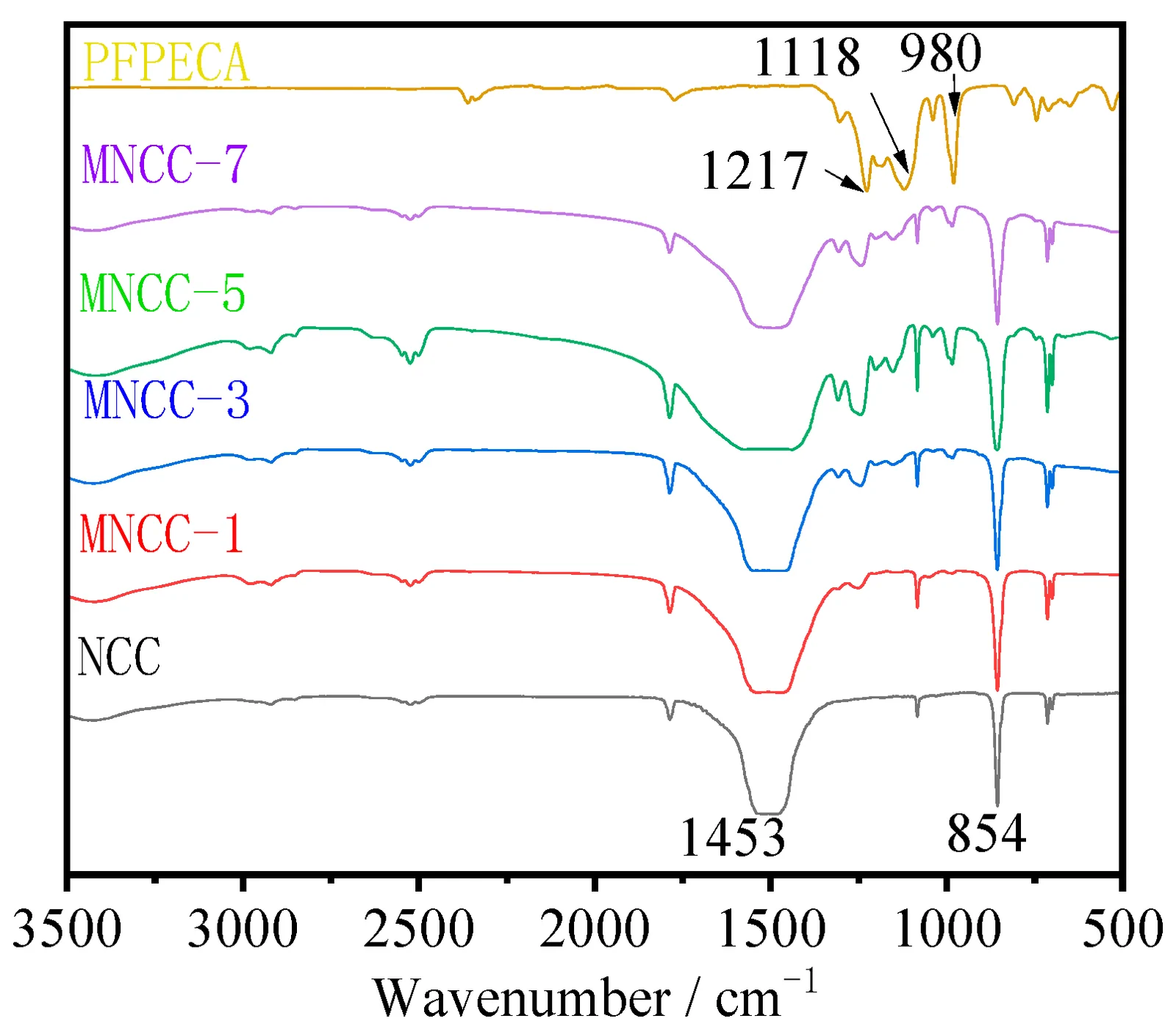
FT-IR spectra of the samples.

Figure 3 presents the FT-IR spectra of the samples, revealing key structural information about the modification process. The PFPECA spectrum exhibits three distinct absorption peaks at approximately 1217, 1118, and 980 cm^−1^, corresponding to CF_3_, C-F near COOH, and other C-F stretching vibrations, respectively [23,27,28]. The NCC spectrum shows characteristic peaks at 1453 cm^−1^ and 854 cm^−1^, which are indicative of aragonite whiskers [12]. After modification, the MNCC spectrum exhibited three characteristic PFPECA peaks (appearing in order: 1217 cm^−1^ peak from MNCC-1, 1118, and 980 cm^−1^ peak from MNCC-3), albeit with reduced intensity, at least confirming that PFPECA molecules successfully attached to the NCC surface. Interestingly, the NCC spectrum lacks the hydroxyl group absorption peak typically observed at approximately 3000–3500 cm^−1^, which contrasts with previous studies [29]. This absence indicates that, if any chemical reaction occurs, the modification reaction occurs at certain active sites on the NCC surface, forming certain chemical bonds rather than proceeding through hydroxyl group interactions, as previously suggested. This finding provides new insights into the mechanism of PFPECA modification of NCC surfaces.

**Figure 4:**
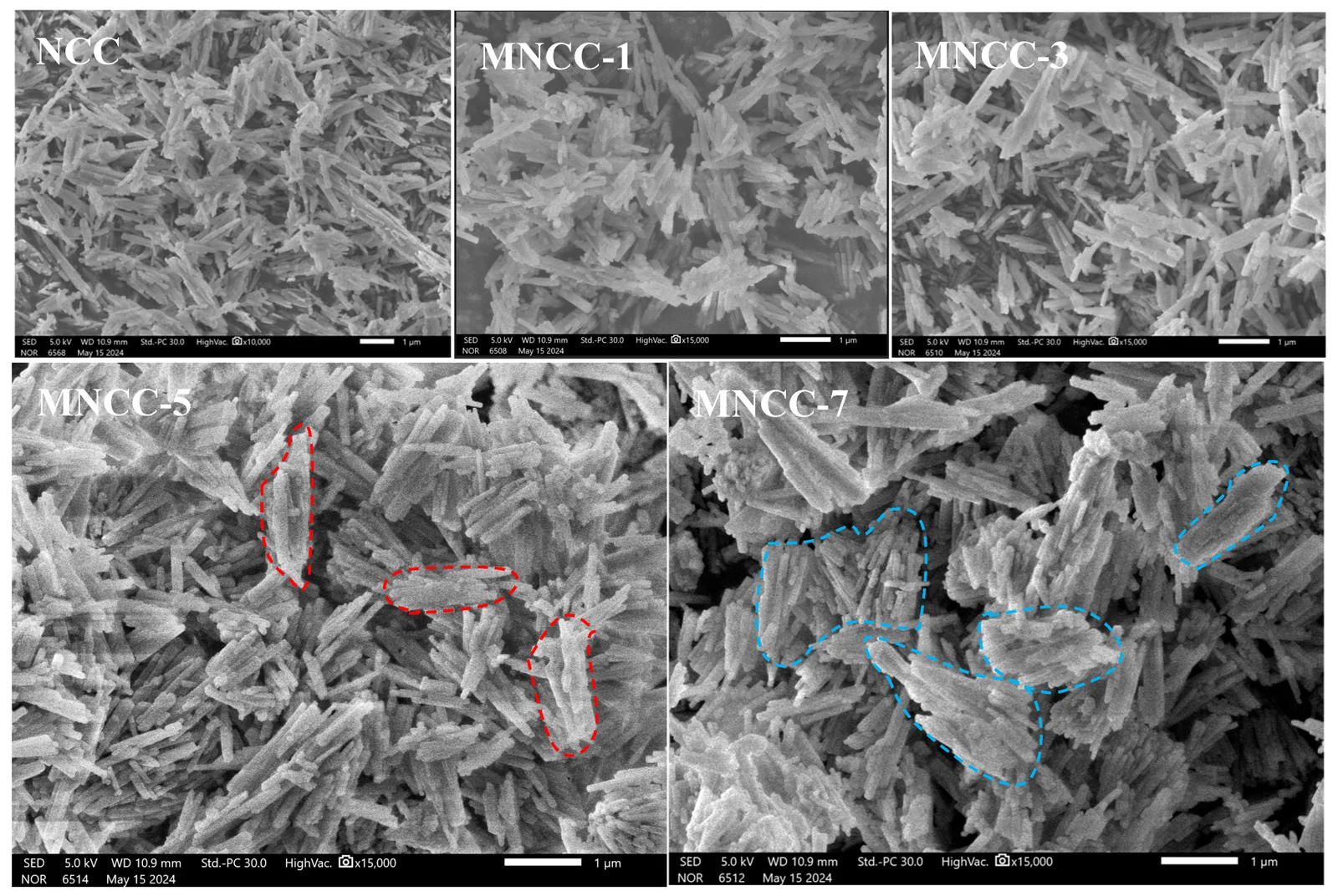
SEM images of NCC and MNCC.

SEM images of the samples are presented in Figure 4. Nanocrystalline whisker are a rod-shaped nanomaterial with diameter ranging from 100 to 150 nm and lengths of 0.5 to 2 μm. They are randomly distributed. At low modifier concentrations, as observed for the MNCC-1 and MNCC-3 samples, the morphology remained similar to that of unmodified NCC. However, when the modifier content reaches or exceeds 5 wt%, a noticeable change occurs. The rod-like MNCC whiskers begin to aggregate along their axes, as highlighted by the red circle in the MNCC-5 sample and the blue circle in the MNCC-7 sample. This aggregation phenomenon can be attributed to the hook-like connection of the big molecules of the modifier (Figure S1) on the NCC surface. The elemental compositions of the powder samples (≤75 µm), as determined by XPS, are presented in Table S1. In theory, if we consider only the molecular formula of calcium carbonate (CaCO_3_), the atomic molar percentages should be 20 mol% for calcium (Ca), 20 mol% for carbon (C), and 60 mol% for oxygen (O). However, the XPS results reveal a different distribution: 20.67 mol% for Ca, 22.31 mol% for C, and 57.02 mol% for O. The slight discrepancies between these experimental results and the theoretical expectations can be attributed to the actual crystal structure of the samples, which influences the elemental distribution in a more complex manner than the simple molecular formula would suggest. The XPS analysis of the samples’ elemental compositions reveals a bias toward surface atoms. This is because the high-energy X-rays are more likely to excite outer electrons of atoms on the surface, leading to the emission of photoelectrons that are then detected by the detector. In this research, nanocrystalline whiskers were identified as aragonite, their structure and morphology are illustrated in Figure S2a based on previous research [30]. Aragonite typically forms rod-like structures. Within this crystal arrangement, calcium (Ca) atoms are positioned on the surface of the crystal unit cell, while oxygen (O) and carbon (C) atoms are only partially exposed on the surface, with the remainder situated within the crystal interior. This structural configuration explains why the percentage of Ca atoms detected is basically consistent with that in the theoretical calculations. Conversely, the measured percentages of O and C atoms are lower or slightly higher than expected due to their partial internalization within the crystal structure.

**Figure 5:**
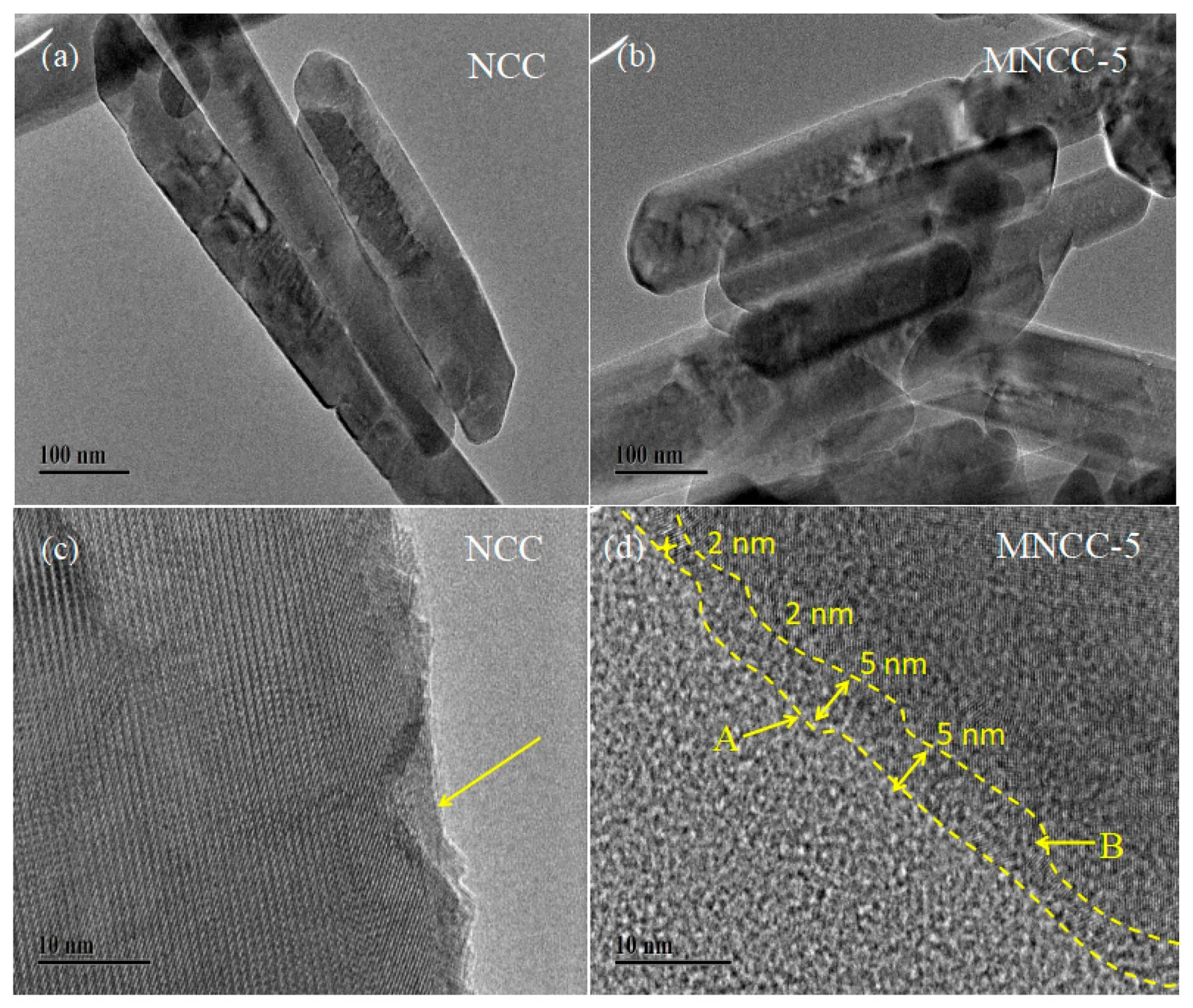
HR-TEM images of NCC and MNCC-5 in the interfacial area.

To clearly observe the surface morphology of the NCC and MNCC samples, HR-TEM was used to observe the samples. The TEM images of NCC and MNCC-5 are shown in Figure 5. The rod-shaped morphology is clearly exhibited in both the two samples (Figure 5a,b). The lattice structure of NCC is also clearly visible (Figure 5c). Upon modification, this lattice structure becomes slightly less distinct (Figure 5d), likely due to the presence of a PFPECA film on the surface. At the interface, a modified layer measuring 2 to 5 nm in thickness is clearly visible, as highlighted by the yellow region in the MNCC-5 sample (Figure 5d).

**Figure 6:**
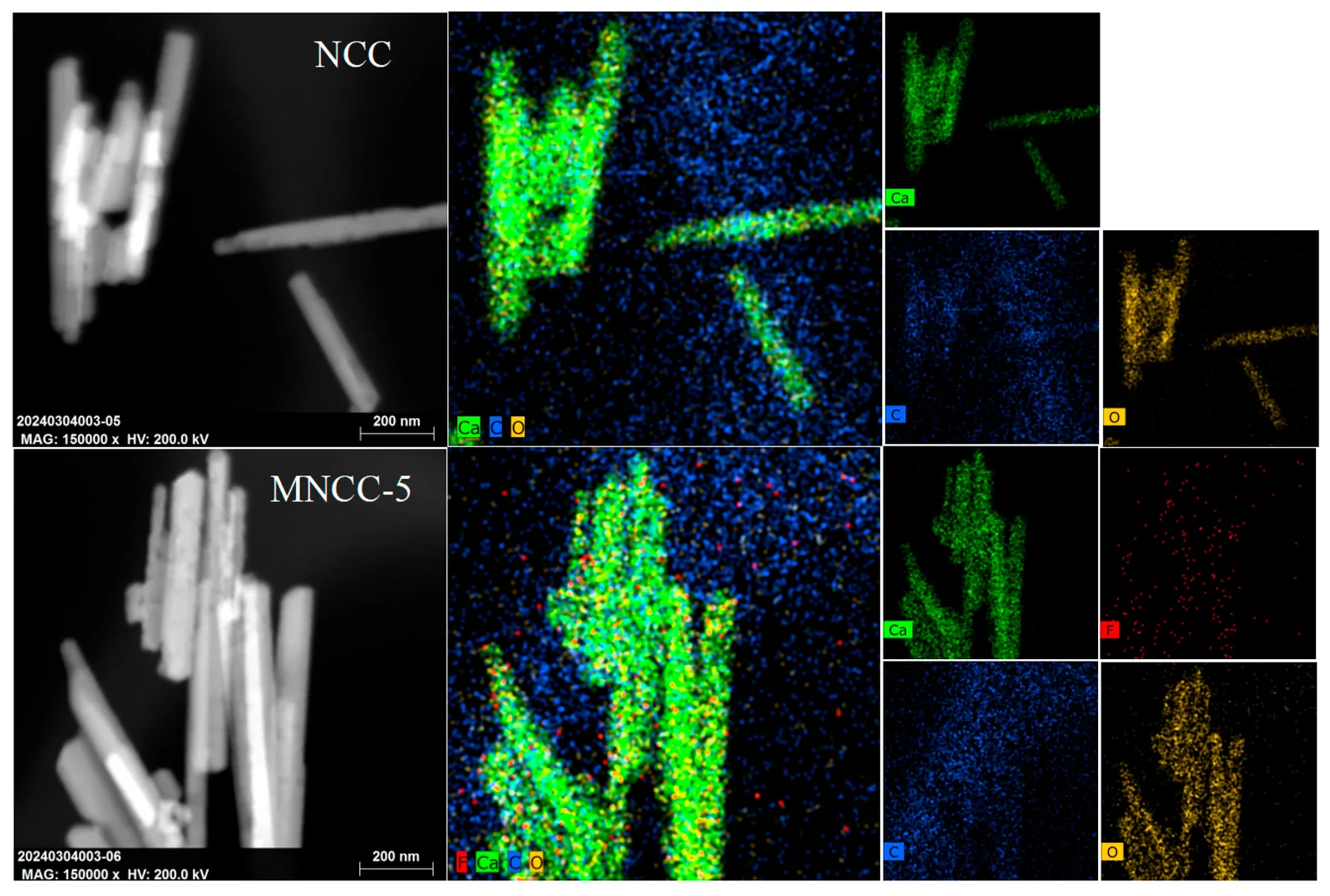
TEM images and map images of NCC and MNCC-5.

Figure 6 displays both TEM images and elemental maps of the NCC and MNCC-5 samples. The elemental maps reveal that Ca, O, and C in NCC, as well as the newly added F in MNCC-5, appear as bright region with the same shape as the sample, except for the C element. Notably, the presence of fluorine in the MNCC-5 sample area provides evidence that the NCC surface was successfully coated with PFPECA. The above analyses have shown that PFPECA molecules are at least successfully coated on the NCC surface.

### 2.3. Surface Modification Mechanism

To explore the modification reaction mechanism on the NCC surface, NCC and MNCC-5 were characterized by XPS. As shown by the FT-IR spectra in Figure 3, no hydroxyl adsorption peaks (3000–3500 cm^−1^) are abserved, indicating that the modification reaction could not occur through interactions between modifier molecules and hydroxyl groups on the NCC surface.

**Figure 7:**
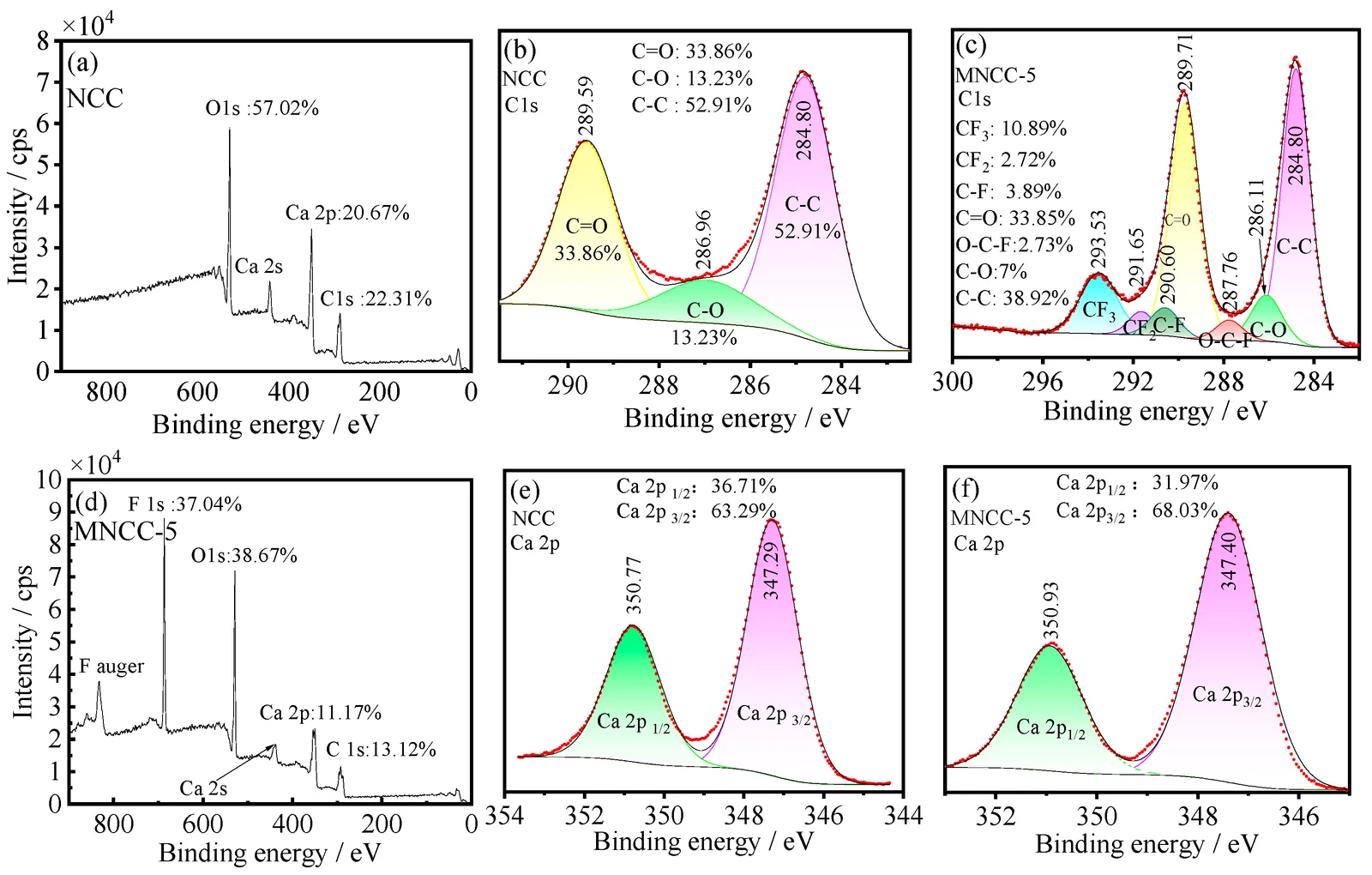
XPS survey scan spectra of NCC (**a**) and MNCC-5 (**d**), high resolution spectra of C1s for NCC (**b**) and MNCC-5 (**c**), Ca 2p for NCC (**e**) and MNCC-5 (**f**), respectively.

Figure 7 shows the XPS spectra of NCC and MNCC-5. Figure 7a,b indicate the full scale and C1s spectra of NCC. The emergence of the F1s peak in Figure 7c,d fully explains that the PFPECA molecules have been successfully incorporated into NCC. The Ca 2p atom ratio obviously decreases from 57.02 mol% in NCC (Figure 7a) to 11.17 mol% in MNCC-5 (Figure 7d), indicating that the modifier molecules cover the Ca atoms. The C1s atomic molar ratio decreases from 22.31 mol% in NCC to 13.12 mol% in MNCC-5, far less than its molar ratio in PFPECA (29.51 mol%, calculated from molecular composition of C_18_O_7_F_35_H) and that in NCC (22.31 mol%) in NCC in Figure 7a. This indicates that modifier molecules also cover the C sites on the NCC surface. Furthermore, the PFPECA molecules should exist on the NCC surface in a standing orientation rather than a laying orientation. Only in this way will the large number of F atoms present on the surface lead to a C atom molar ratio in MNCC-5 lower than that in the modifier and NCC. The O1s atomic molar ratio decreases from 57.02 mol% in NCC to 38.67 mol% in MNCC-5, obviously, the modifier molecules have also covered the O sites on the NCC surface. Overall, after surface modification, the molar percentage of Ca, C and O greatly decreased. A possible reason is as follows: the XPS only tests the surface atom information of materials and has obvious difference in surface depth. about 2–5 nm for inorganic materials and 4–10 nm for organic materials. For these composites consisting of an organic layer coated on an inorganic bulk, the atom photoelectronic information excited from the matrix NCC rapidly attenuates to various extents due to the shielding effect of the above organic layer. Although modifier molecules cover the Ca, C and O sites on the NCC surface, the modification reaction possibly takes place on the Ca sites rather than the C and O sites. To clarify the specific reaction mechanism, the narrow scan spectra of C1s and Ca 2p were split into multiple small peaks for further analysis.

Figure 7b,c show that the C=O group ratio shows nearly no any change before (33.86%) and after (33.85%) modification. This also supports the above analysis results. Comparing Figure 7e,f, obviously, surface modification makes the binding energy of Ca 2p 1/2 and Ca 2p 3/2 slightly increase from 350.77 eV and 347.29 eV to 350.93 eV and 347.40 eV, respectively, which demonstrates that the modification reaction occur at the Ca site on the NCC surface. More precisely, the COO^−^ group of the PFPECA anion reacts with the Ca^2+^ on the NCC surface and forms the ionic bonds. Including the original two O atoms near the Ca atom (Figure S2a), there are three oxygen atoms around the Ca atom, therefore, its binding energy slightly increases. The only two positive charges in the Ca atom casuse the electron cloud of the O-C bond to shift toward the the C=O of NCC and the COO^−^ group of PFPECA; thus, after modification, the binding energy of the C-O bond decreases from 286.96 eV to 286.11 eV, and that of the C=O bond increases from 289.59 eV to 289.71 eV (Figure 7b,c).

**Figure 8:**
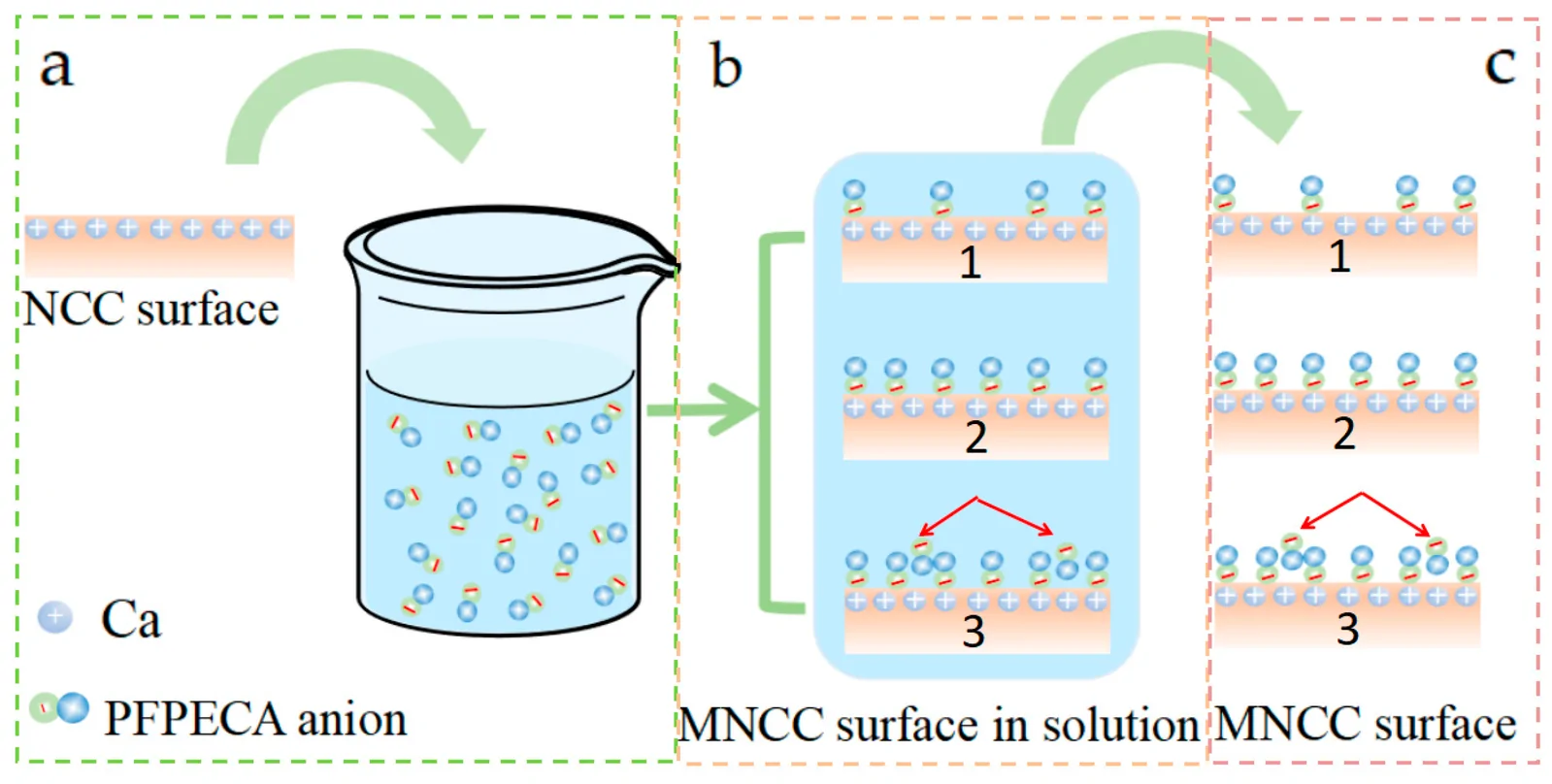
Modification mechanism diagram of NCC by PFPECA. (**a**) NCC and modifier solution, (**b**) Surface modifcation process, (**c**) MNCC surface.

Based on the above analysis, we propose the following modification reaction mechanism. Figure 8 illustrates the reaction mechanism. The whole process is divided into three steps: First, in the experimental solution, PFPECA reacted with equimolar NaOH to form a modifier solution containing PFPECA anion with a terminal COO^−^ groups and Na^+^, and then NCC was added to this solution (Figure 8a); second, the COO^−^ groups of the PFPECA anion bonded to the Ca^2+^ sites on the NCC surface, with the molecular chain composed of CF_2_ and CF_3_ groups oriented outward (1 in Figure 8b). As the PFPECA concentration increased, more PFPECA anions bonded with Ca^2+^ and covered a larger surface area of NCC, forming a modifier layer (2 in Figure 8b). When the number of PFPECA anions was sufficiently large, the large molecular structure of PFPECA shielded some Ca atoms on the surface, resulting in the remaining area being so small that the COO^−^ groups could not approach the surface of NCC and were only orientated randomly, for example, oriented outword (red arrow in 3 of Figure 8b). Thus, although the modifier layer on the NCC surface was denser, the hydrophobicity decreased due to the COO^−^ groups facing outward. Finally, when the modification reaction finished, the MNCC was filtered and dried, obtaining a series of MNCC samples (1, 2 and 3 of Figure 8c) according to the modifier mass ratio.

This explains the observed shift in the surface zeta potential from positive to negative, as depicted in Figure 1a. The resulting MNCC exhibits amphiphobic properties, meaning that it is both hydrophobic and oleophobic. This dual repellency is attributed to the low surface energy imparted by the PFPECA film coating the NCC surface [31].

### 2.4. Coverage Ratio of MNCC

**Figure 9:**
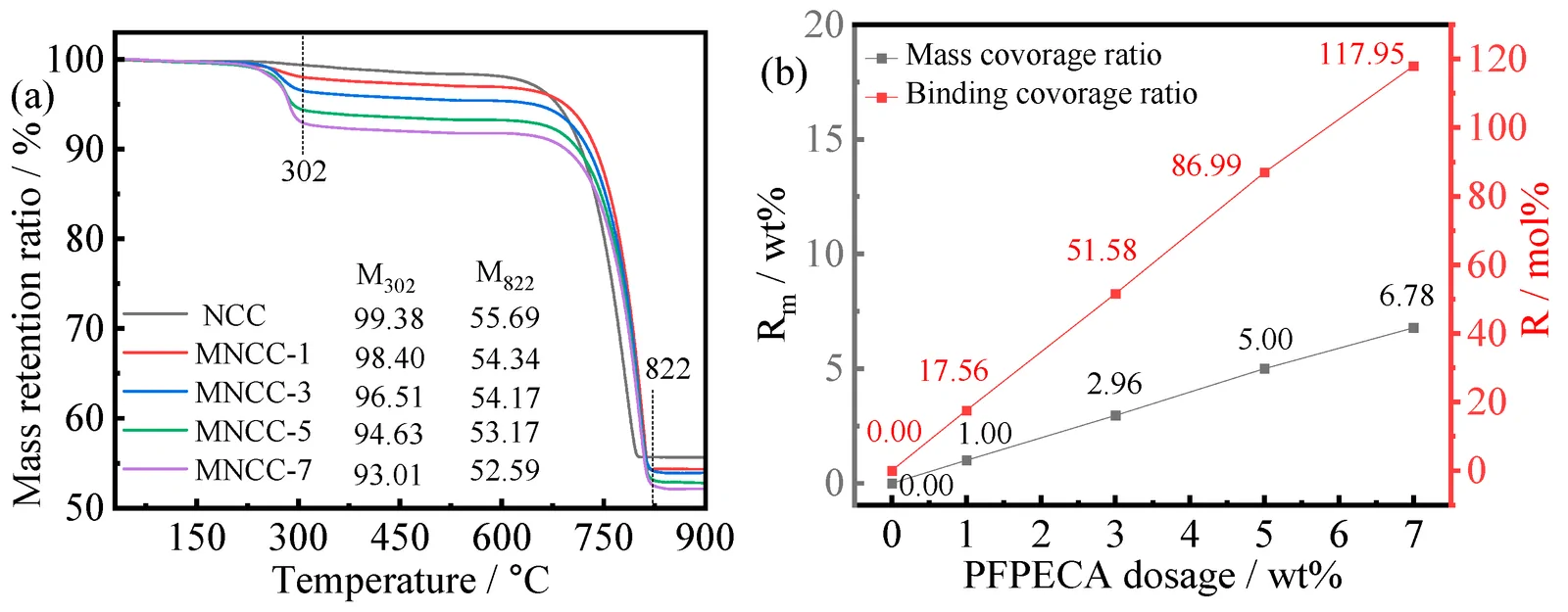
TG curves (**a**), mass and binding coverage ratio vs. PFPECA dosage curves (**b**) of samples.

The coverage amount of modifier on the NCC surface is also a key factor determining the modification effect. This can also be evidenced by the active ratio change in Figure 1 and the zeta potential change in Figure 2. However, in the experiment, the dosage of PFPECA was only the designed dosage relative to the NCC mass. It was dissolved in water containing equimolar NaOH to form PFPECA anions. To obtain the practical coverage mass of modifier on the NCC surface, the NCC and MNCCs were analysed by TG-DTG technique. As shown in Figure 9a, two TG regions appear in the temperature ranges of 200 to 302 °C and 650 to 822 °C, which are attributed to the volatilization of water and modifier molecules, and the decomposition of CaCO_3_. The mass retention ratios, M_302_ and M_822_ are mass retention at 302 °C and 822 °C, respectively. The PFPECA mass (M_PFPECA_), CaCO_3_ mass (M_CaCO3_) and mass coverage ratio (R_m_) can be calculated using Equations (S1)–(S3) (refer to Supplementary Files). However, what truly reflects the degree of modification should be the bonding coverage ratio, namely, R = N_PFPECA_/N_Ca_, Here, N_PFPECA_ and N_Ca_ are the molar numbers of PFPECA and Ca atoms on the surface of MNCCs. In other words, it respresents the proportion of modifier molecules that reacted with Ca^2+^ ions on the NCC surface to the total number of calcium ions on the NCC surface. Their calculation methods refer to Equations (S4)–(S6) (Supplementary Files). Figure 9b indicates that the mass coverage ratio (R_m_) and binding coverage ratio (R) increase with increasing PFPECA designed dosage. R_m_ is basically close to the designed dosage and slightly less than that; for example, PFPECA designed dosages are 3 wt% and 7 wt%, while R_m_ are 2.96 wt% and 6.37 wt%, respectively. The change in R is dramatic; when the PFPECA dosage is 5 wt%, R reaches 86.99 mol%, indicating that the large majority of Ca is bound to the PFPECA anion. Further increasing the designed modifier dosage results in an R value of up to 117.95 mol%. Evidently, some excess modifier molecules have no chance to react with Ca^2+^ on the NCC surface due to the molecular spatial configuration of the modifier (refer to Figure S1; PFPECA molecules appear as a linear cluster rather than existing as a linear molecular configuration). Therefore, they are merely entangled with the PFPECA molecules that have already undergone the modification reaction with NCC and remain on its surface. Thus, its COO^−^ groups will orient randomly and may face outward. In general, considering from the viewpoint of binding coverage ratio, a 5 wt% PFPECA dosage is conducive to the modification effect; this result is consistent with the previous study results about the active ratio in Figure 1a and the zeta potential in Figure 2a. It is also completely consistent with the previous modification analysis in Figure 8.

### 2.5. Effect of Surface Modification on the Physical Properties of NCC

**Figure 10:**
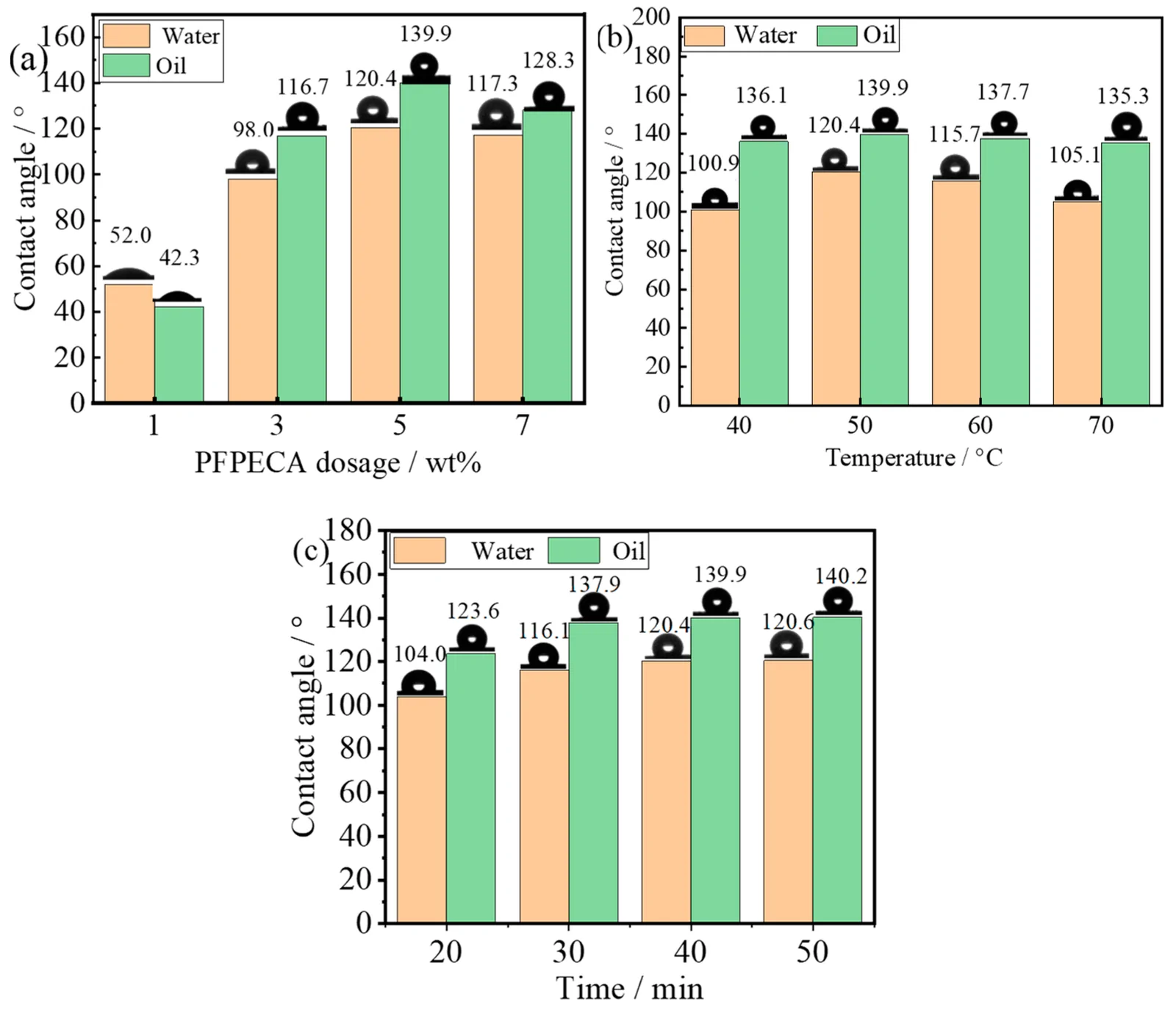
Water and oil contact angles (**a**–**c**) of MNCCs.

This study aim to impart hydrophobicity and oleophobicity to NCC through surface modification. Figure 10a–c show the water and oil contact angles of the MNCCs obtained under various conditions. The MNCCs exhibited exceptional hydrophobicity and oleophobicity due to PFPECA modification. As the PFPECA dosage increased, both the water and oil contact angles initially increased and then decreased, reaching peak values at 5 wt% PFPECA: 120.4° for water and 139.9° for oil (Figure 10a). This behavior is clearly attributed to the CF_2_ and CF_3_ groups in the PFPECA molecules, which reduce surface tension and provide the lowest surface free energy among solid surface [31,32,33]. The modification reaction imparted water- and oil-repellent properties to the NCC. Furthermore, fluorinated material films offer additional advantages, including high thermal, chemical, and weather resistance [34,35]. When the PFPECA dosage exceeds 5 wt%, the water and oil contact angles decrease, as previous analysis in Figure 8 and Section 2.3, likely because surplus terminals COO^−^ groups of the PFPECA molecule face outward. Using 5 wt% PFPECA as the optimal dosage, the effects of temperature and time on the water and oil contact angles were also investigated. Figure 10b,c reveal that the optimal temperature and time for the modification process are 50 °C and 40 min, respectively. This results is also consistent with the active ratio results in Figure 1.

**Figure 11:**
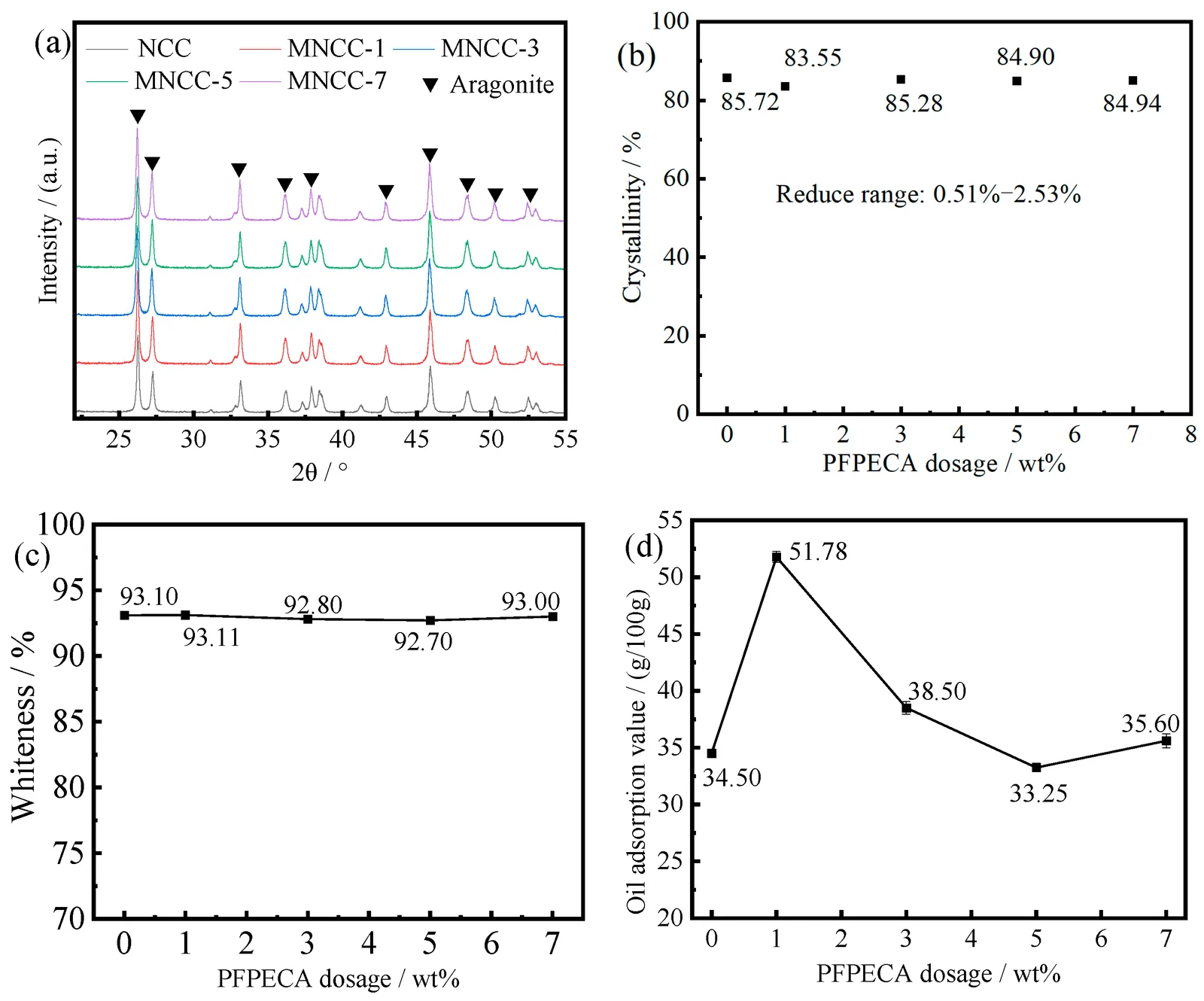
(**a**) XRD pattern of the samples, effect of modifier dosage on the crystallinity (**b**), Oil adsorption values (**c**) and whiteness (**d**) of NCC.

Figure 11 shows the impact of PFPECA modification on various properties of NCCs. The aragonite crystal structure of both NCC and MNCC are confirmed in Figure 11a. Figure 11b shows that NCC is not pure aragonite and has 85.72% crystallinity, with the remainder being amorphous. After modification, the crystallinity slightly decreased to 83.55–85.28%, a minor reduction of 0.51–2.53% due to the surface modifier film. The whiteness remains largely unchanged, as shown in Figure 11c. These findings suggest that surface modification minimally affects the aragonite crystal structure and whiteness. The influence of PFPECA modification on oil absorption is depicted in Figure 11d. Oil absorption initially increases with increasing PFPECA dosage and then decreases. At a PFPECA dosage of 1 wt%, the total oil adsorption value increases from 34.50% to 51.78%. Further dosage increases led to a gradual reduction in oil absorption, reaching a minimum of 33.25% at the 5 wt% dosage before slightly increasing to 35.60% at the 7 wt% dosage. Interestingly, despite the oleophobic properties of MNCC, the mechanical forces involved in oil absorption testing still enable oil to penetrate into the micropores of the sample.

Table 1 provides detailed information on the SSA and micropore characteristics of the samples. Unlike conventional nanomaterials, the oil absorption capacity of these modified samples does not show a positive correlation between oil absorption capacity and either SSA or pore volume. This deviation is attributed to the influence of the surface modifier film. Instead, the oil absorption capacity is primarily linked to the peak aperture size of the micropores. Larger micropores demonstrate a greater ability to absorb oil molecules when mechanical force is applied during measure process. This relationship is exemplified by the contrasting behaviors of MNCC-5 and MNCC-1. MNCC-5, with its smaller peak aperture of 2.03 nm, exhibited the lowest oil absorption at 33.25%. Conversely, MNCC-1, possessing a larger peak aperture of 2.41 nm, demonstrated the highest oil absorption capacity at 51.78%. These findings highlight the significant role of micropore size in determining the oil absorption properties of these modified materials.

Table 2 lists some literatures on modified NCCs or their applications. Many reports about describe hydrophobic products obtained after modification. These products can be applied in many fields such as building conservation, soil protection, foam stabilization, polymer composites and papermaking. However, seldom studies have reported MNCC with amphiphobic properties In this study, a novel approach was employed to simultaneously endows NCCs with amphiphobic properties. This approach has potential application in self-cleaning coatings, anti-fouling, and anti-adhesion.

## 3. Materials and Methods

### 3.1. Materials

**Figure 12:**
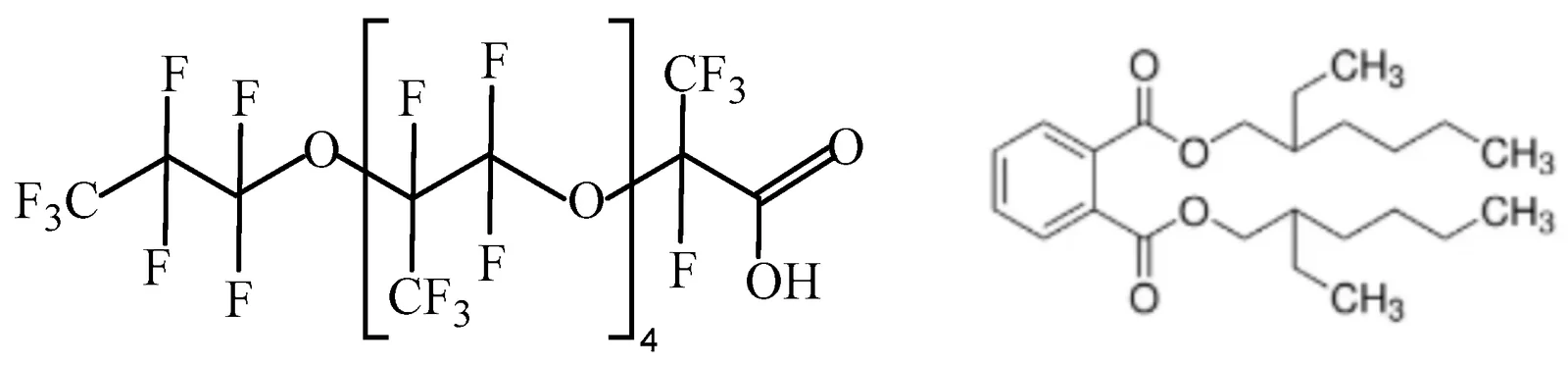
Molecular structure of PFPECA (**left**) and DP (**right**).

The NCC used in this study was sourced from Shanxi Lvliang Shiqi Shidai New Material Co. Ltd., Lvliang, China, and had a surface area of 12.93 m^2^/g. Its nanosize characteristics will be detailed in subsequent sections. PFPECA, sodium hydroxide (NaOH) and dioctyl phthalate (DP) were acquired from Shanghai Maikelin Biochemical Technology Co. Ltd., Shanghai, China. PFPECA has a molar mass of 994 g/mol, and its molecular structure is illustrated in Figure 12. The molecular structure of DP is also shown in Figure 12.

### 3.2. Modification of NCC

A predetermined amount of PFPECA, proportional to the NCC mass, was dissolved in water. The solution temperature was raised to 40 °C, followed by the addition of NaOH at a 1:1 molar ratio to PFPECA. NCC was then gradually introduced into this solution under constant stirring, maintaining a 1:5 NCC-to-water mass ratio. The modification reaction conditions were optimized step by step. The PFPECA dosage was set at 1 wt%, 3 wt%, 5 wt% and 7 wt% based on the NCC mass. The reaction time was set at 40 min, 50 min, 60 min and 70 min. After completion, the product was filtered and dried to obtain MNCC. The resulting MNCCs were designated using the following nomenclature: MNCC-n, here, n represents the mass percentage to NCC mass. For example, MNCC-7 represents the modified NCC obtained in the solution containing 7 wt% PFPECA.

### 3.3. Characterization

Fourier transform infrared (FT-IR) spectroscopy was performed on a Perkin-Elmer 1730 spectrometer (Perkin Elmer Corporation, Norwalk, CT, USA) using KBr pellets. X-ray diffraction (XRD) patterns were obtained using a Bruker D2-Phaser advance diffractometer (Bruker AXS SE, Karlsruhe, Germany) with Cu Kα radiation (λ = 0.15418 nm) and scanning from 10° to 80° with a 0.02° step size. The microstructure were observed using a JSM-IT500HR scanning electron microscope (SEM) (JEOL Ltd., Akishima, Japan). Thermogravimetric (TG) analysis was conducted on a Mettler Toledo TGA/DSC3+ analyzer (Mettler Toledo AG, Schwerzenbach, Switzerland) from 20 to 900 °C at 20 °C/min under a N_2_ atmosphere. High-resolution transmission electron microscopy (HR-TEM) was performed using a JEM-2100F microscope (JEOL Ltd., Akishima, Japan) to examine the nanostructure morphology. The specific surface area (SSA) was measured by nitrogen adsorption using the BET method on an AutoSorb (BK300) apparatus (JWGB Instrument Co., Ltd., Beijing, China). The zeta potential was determined using a Zetasizer Nano ZS90 Analyzer (Malvern Panalytical, Malvern, UK). The whiteness of samples was measured according to the national standard [41] for inorganic chemical products. Water and oil contact angles of samples were assessed using a Kruss contact angle tester (Kruss GmbH, Hamburg, Germany). The active ratio was determined as described in our previous publication [42]. Oil adsorption was tested using DP following the National Standard for calcium carbonate [43]. All measurements were performed in triplicate, and the results are reported as the means. X-ray photoelectron spectroscopy (XPS) was performed on an X-ray photoelectron spectrometer (Axis Ultradld, Kratos Analytical Limited, Manchester, UK). The test conditions were as follows: Al Kα X-ray were used as the source (*hv* = 1486.6 eV). Sample analysis region: 700 µm × 300 µm, power/90 W, full scan pass energy/160 eV, step length/1.0 eV; narrow scans pass energy/20 eV, step length/0.05 eV, the base vacuum/5 × 10^9^ Pa. Electron binding energy was calibrated using the main C1s peak (284.8 eV).

## 4. Conclusions

This study successfully synthesized and characterized amphiphobic NCCs modified with PFPECA, providing significant insights into the surface modification mechanism and resulting properties. The optimized process (5 wt.% PFPECA, 50 °C, 40 min) yielded MNCC with superior amphiphobic properties, demonstrating water and oil contact angles of 120.4° and 139.9°, respectively. FT-IR analysis revealed an absence of hydroxyl groups on unmodified NCC, leading to a novel understanding of the modification mechanism: COO^−^ groups from PFPECA form Ca-O ionic bonds with Ca sites on the NCC surface. HR-TEM revealed an exceptionally thin modifier film (2–5 nm) that significantly altered the surface properties of NCC. Comprehensive characterization confirmed that the modification process minimally impacted the fundamental properties of NCC, with only a slight decrease in crystallinity (0.51–2.53%). The oil absorption properties were found to be influenced primarily by the peak aperture size of micropore rather than by the specific surface area or pore volume. These findings have significant implications for developing self-cleaning coatings and amphiphobic surfaces without altering the core properties of NCC. This research advances understanding of NCC surface modification mechanisms and presents a novel approach for creating highly amphiphobic materials with potential industrial applications.

## Figures and Tables

**Table 1:** SSA of NCC and MNCC.

Samples	BET SSA (m^2^/g)	Pore Volume (cm^3^/g)	Peak Aperture nm
NCC	12.93	0.0750	2.120
MNCC-1	12.61	0.0700	2.410
MNCC-3	13.61	0.0620	2.400
MNCC-5	14.29	0.0720	2.030
MNCC-7	14.79	0.0920	2.400

**Table 2:** Comparasion between this study results and previous literatures.

Samples	Modifer	Water Angle/°	Oil Angle/°	Application Field	Ref.
Limestone and marble	Silicic acid ester/modifed CaCO_3_ with ethanol solution	>150		Building conservation	[36]
sandstones	Perfluorodecyl-triethoxy silane ethanol solution containing nano CaCO_3_	149	-	Protecting red sandstone and again acid rain	[37]
CaCO_3_	In-situ modification during precipitation process by adding alkanolamide 6502(ALK)	159.2	-	Enhance the utilization of Ca in seawater	[20]
Nano CaCO_3_	Anionic fluorinated surfactant (Zonyl TBS)	115.1	-	As foam stabilizer	[38]
Nano CaCO_3_	NaS adding into synthetic solution of Nano CaCO_3_	127	-	In polymer composites	[39]
Nano CaCO_3_	Sodium oleate solution	112	-	Coating paper	[40]
Nano CaCO_3_	PFPECA solution containg equal molar NaOH	120.4	139.9	Anti-fouling	This study

## Data Availability

The data is available upon request from the corresponding author.

## Supplementary Materials

The following supporting information can be downloaded at: https://www.mdpi.com/article/10.3390/molecules31183320/s1, Figure S1: molecular structure model of PFPECA anion; Figure S2: (a) Crystal structure and (b) morphology of the NCC (aragonite) (**c**) crystalline cellulose; Table S1: Atom mol percentage of the sample surface by XPS test for NCC; Table S2: M_CaCO3_, M_PFPECA_, R_m_ and R of MNCCs.
