# Supplementary material for: A Novel Approach Endowing Nanocalcium Carbonate with Amphiphobic Properties Through Perfluoropolyether Carboxylic Acid Surface Modification: Elucidating the Surface Modification Mechanism
Source: Molecules. 2026 Sep 19;31(18):3320. doi: 10.3390/molecules31183320 (PMC13610372; doi:10.3390/molecules31183320)
Supplement: Supplementary file 1 [file molecules-31-03320-s001.zip › molecules-4471741-supplementary.pdf]

Supplementary files

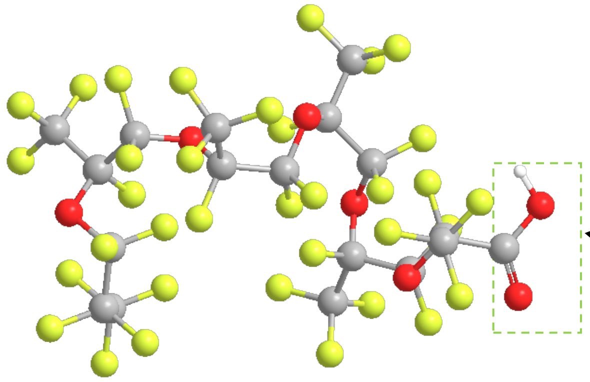

Figure S1. molecular structure model of PFPECA anion
（gray, red, white and yellow ball resperent C, O, H and F atoms, respectively）

Table S1. Atom mol percentage of the sample surface by XPS test for NCC

Samples	C	O	F	Ca
NCC / atom mol%	22.31	57.02	0.00	20.67

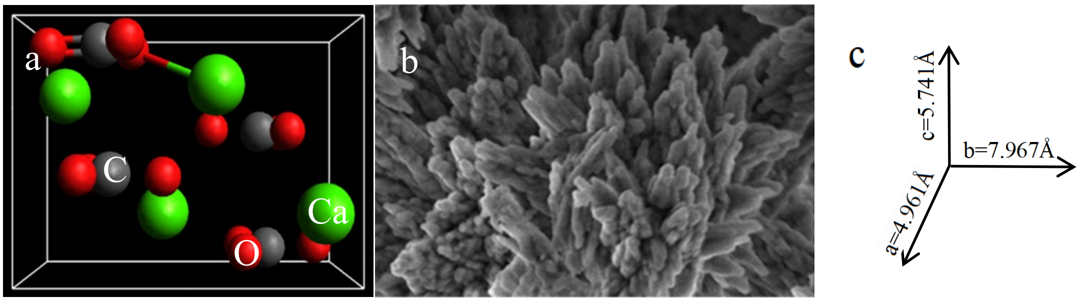

Figure S2. **(a)** Crystal structure and **(b)** morphology of the NCC (aragonite)[30] (Copyright permission obtained from publisher) **(c)** crystalline cellulose, here, a=4.961Å b=7.967Å c=5.741Å

As be shown in Fig.S2a, each aragonite crystal cell contain four $CaCO_3$ molecules. The four Ca atoms are respectively located on four side faces of the crystal cell. Namely, two faces ($F_{a\times c}$) and two faces ($F_{b\times c}$), upper and lower faces ($F_{a\times b}$) still keep certain distance to Ca atom, therefore, we suppose the two faces no surface modification reaction took place due to the Ca atoms no exist on its face. Thus, for each crystal cell on the muticrystal aragonite surface, among three faces, such as $F_{a\times c}$, and $F_{b\times c}$ directly appear on the face of multicrystal cells only have two third chance.$F_{a\times b}$ only has one third chance appear on the surface of aragonite.

Calculation method of Mass covorage ratio (R_m) and binding covorage ratio (R) :

According to the DT curves of NCC and MNCCs, PFPECA mass (M_{PFPECA}), $CaCO_3$ mass (M_{CaCO3}) and mass covorage ratio (R_m) can be calculated by the following

S-equation (S1) to (S3):

$CaCO_3$ (100) → CaO (56) ＋ CO_2 (44) ↑

$$M_{caco3} = \frac{A \times M_{822} \times 100}{56} \quad \text{(S1)}$$

$$M_{PFPECA} = A \times \{1 - M_{302} - (1 - 99.38\%)\} = A \times (99.38\% - M_{302}) \quad \text{(S2)}$$

$$R_m = M_{PFPECA} / M_{caco3} \quad \text{(S3)}$$

Here, A is the total mass of each sample. The molar mass of PFPECA anion is 994, so its molar number is calculated by the following S-equation (S4)

$$N_{PFPECA} = \frac{M_{PFPECA}}{994} \quad \text{(S4)}$$

Based on the common sense, the arogonite crystal (rok-like) is consist of crystal cell, among the axis side four faces, two faces are composed of $F_{a\times c}$, other two faces are composed of $F_{b\times c}$. The two terminal faces of rod-like arogonite are composed of $F_{a\times b}$. As above mention, we suppose the two faces no any surface modification reation take place. On each $F_{a\times c}$ or $F_{b\times c}$ face, there is a Ca atom. Such, the specific surface area (S_{NCC}=12.93m^2/g) of NCC by BET measure, S_{NCC} multipled by M_{caco3}, obtaining surface area of corresponding MNCC. Therefore, the molar number of Ca atom on the surface of MNCC can be calculated by S-equation (S5):

$$N_{Ca} = \frac{2 \times S_{NCC} \times M_{CaCO3}}{(F_{a\times c} + F_{b\times c}) \times N_a} \quad \text{(S5)}$$

Here, $F_{a\times c}$ and $F_{b\times c}$ is crystal face $a\times b$ and $b\times c$, N_a is Avogadro's constant. The binding covorage ratio R can be calculated by S-equation (S6):

$$R = \frac{N_{PFPECA}}{N_{Ca}} \times 100\% \quad \text{(S6)}$$

In equation (S1) to (S6), A, M_{caco3}, M_{PFPECA}, N_{PFPECA}, N_{Ca}, N_a, R_m and R respresant the mass of sample measured, $CaCO_3$ mass, PFPECA mass, PFPECA molar number, Ca atom molar number on the surface of sample measured, Avogadro's constant and modifier mass covorage ratio and binding covorage ratio, respectively.

Table S2 M_{CaCO3}, M_{PFPECA}, R_m and R of MNCCs

Samples	M_{CaCO3}	M_{PFPECA}	R_m / wt%	N_{PFPECA}/mol	N_{Ca} /mol	R / mol%
NCC	0.9945A	0	0.00	0.00	5.754E-5A	0.00
MNCC-1	0.9704A	0.0098A	1.00	9.800E-6A	5.614E-5A	17.56
MNCC-3	0.9673A	0.0287A	2.96	2.887E-5A	5.597E-5A	51.58
MNCC-5	0.9495A	0.0475A	5.00	4.779E-5A	5.494-5A	86.99
MNCC-7	0.9391A	0.0637A	6.78	6.408E-5A	5.433E-5A	117.95
